# Supplementary figures and images for: An insight into the gut microbiota of healthy and allergic West Highland Whiter Terrier dogs
Source: PLoS One. 2025 Aug 27;20(8):e0328100. doi: 10.1371/journal.pone.0328100 (PMC12385407; doi:10.1371/journal.pone.0328100)

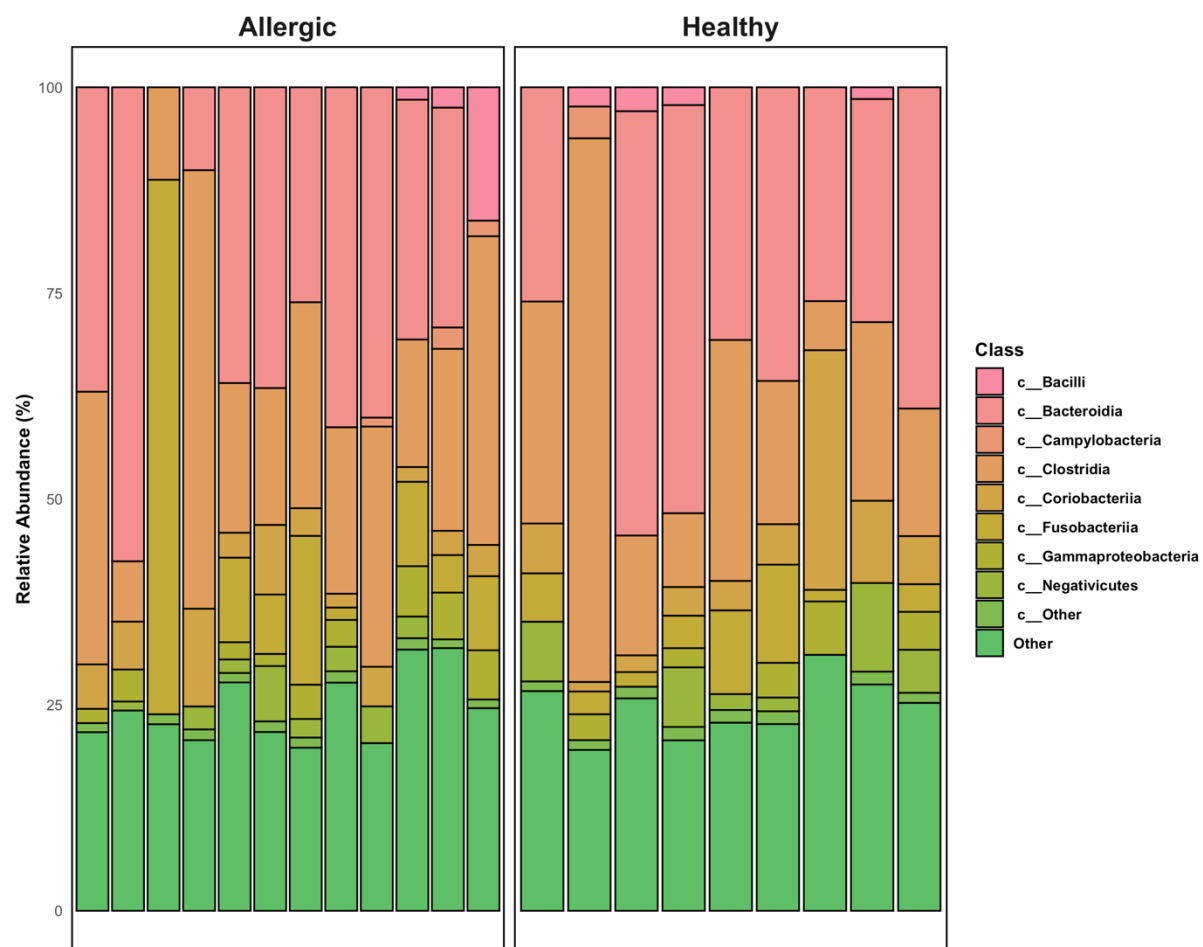

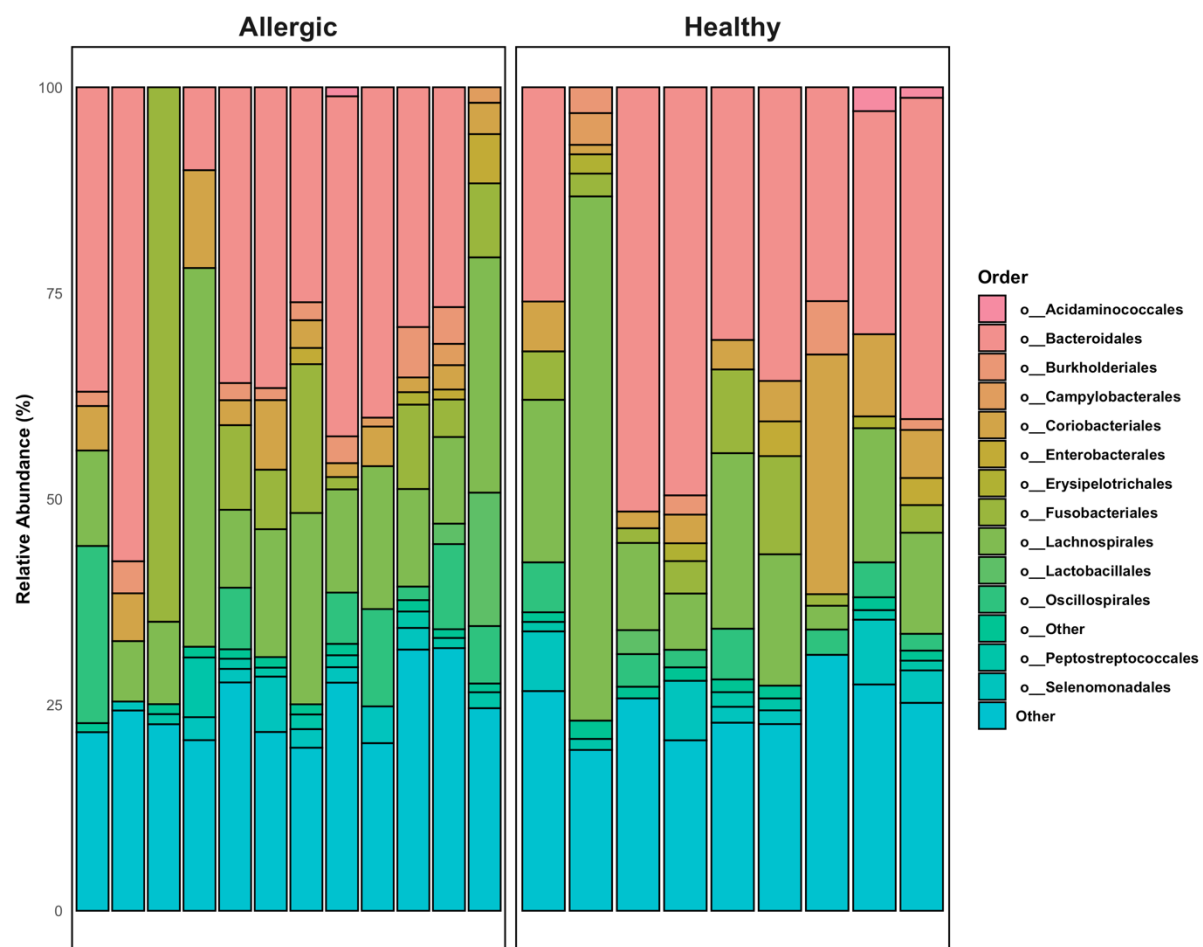

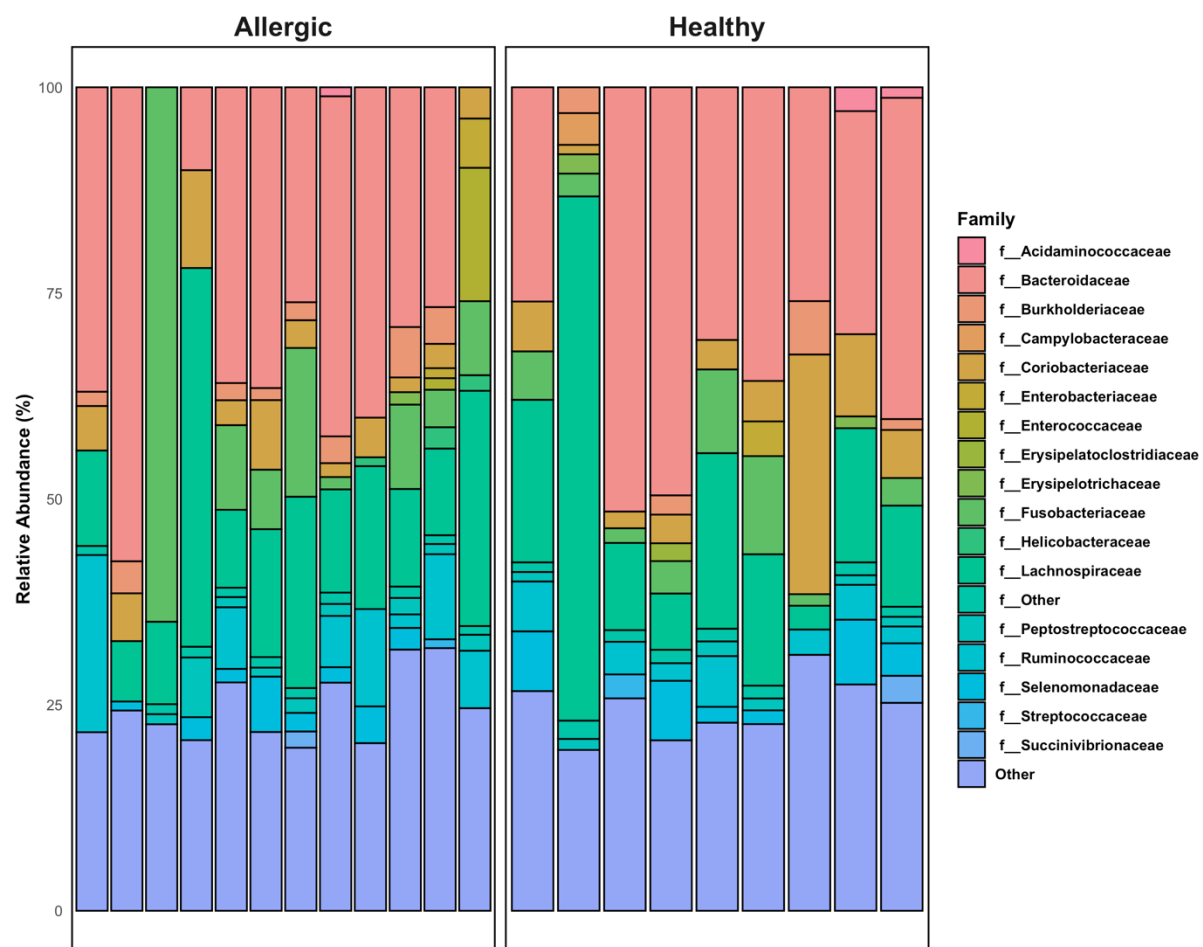

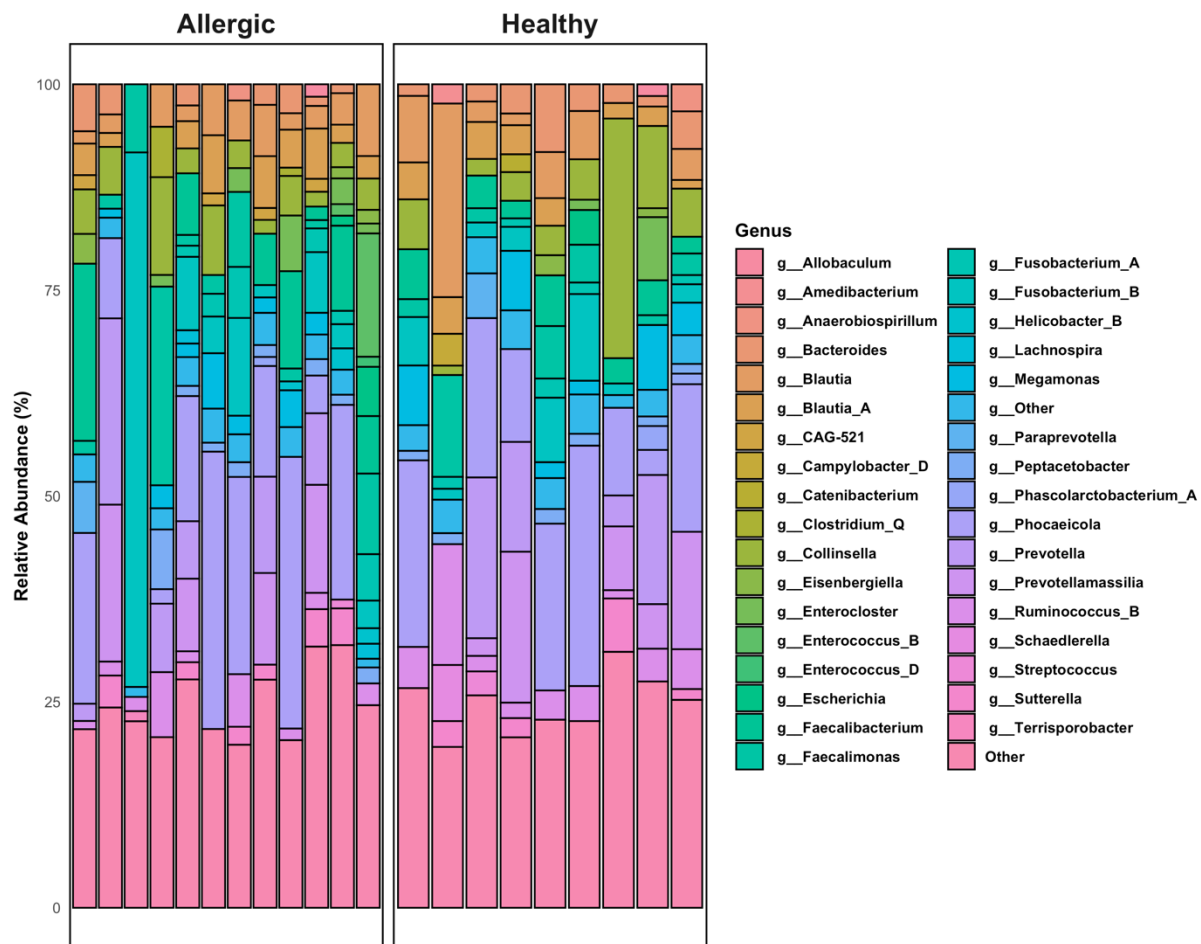

Supplement: S1 Fig — (PDF) [file pone.0328100.s001.pdf]
